# Case Discussion and Literature Review: Cancer Immunotherapy, Severe Immune-Related Adverse Events, Multi-Inflammatory Syndrome, and Severe Acute Respiratory Syndrome Coronavirus 2

**DOI:** 10.3389/fonc.2021.625707

**Published:** 2021-02-04

**Authors:** Dristhi Ragoonanan, Sajad J. Khazal, Rodrigo Mejia, Linette Ewing, Jean-Bernard Durand, Lara Bashoura, Jean Tayar, Natalie Dailey Garnes, Demetrios Petropoulos, Priti Tewari, Micah Bhatti, Ali Haider Ahmad, Jose Cortes, Shehla Razvi, Katrina McBeth, Rita Swinford, Basirat Shoberu, Waseem Waseemuddin, Linda Chi, Jonathan B. Gill, Wafik Zaky, Najat Daw, Cristina Gutierrez, Welela Tereffe, Partow Kebriaei, Katayoun Rezvani, Elizabeth J. Shpall, Richard E. Champlin, Kris M. Mahadeo

**Affiliations:** ^1^ Pediatric Stem Cell Transplantation and Cellular Therapy, CARTOX Program, University of Texas at MD Anderson Cancer Center, Houston, TX, United States; ^2^ Pediatric Critical Care Medicine, University of Texas at MD Anderson Cancer Center, Houston, TX, United States; ^3^ Department of Cardiology, University of Texas at MD Anderson Cancer Center, Houston, TX, United States; ^4^ Department of Pulmonary Medicine, University of Texas at MD Anderson Cancer Center, Houston, TX, United States; ^5^ Department of Rheumatology, University of Texas at MD Anderson Cancer Center, Houston, TX, United States; ^6^ Department of Infectious Disease, University of Texas at MD Anderson Cancer Center, Houston, TX, United States; ^7^ Department of Pathology, University of Texas at MD Anderson Cancer Center, Houston, TX, United States; ^8^ Department of Pediatric Pulmonary Medicine, University of Texas at MD Anderson Cancer Center, Houston, TX, United States; ^9^ Department of Pediatric Nephrology, University of Texas at MD Anderson Cancer Center, Houston, TX, United States; ^10^ Department of Neuroradiology, CARTOX Program, University of Texas at MD Anderson Cancer Center, Houston, TX, United States; ^11^ Department of Pediatric Oncology, University of Texas at MD Anderson Cancer Center, Houston, TX, United States; ^12^ Department of Critical Care Medicine, CARTOX Program, University of Texas at MD Anderson Cancer Center, Houston, TX, United States; ^13^ University of Texas at MD Anderson Cancer Center, Houston, TX, United States; ^14^ Stem Cell Transplantation and Cellular Therapy, CARTOX Program, University of Texas at MD Anderson Cancer Center, Houston, TX, United States

**Keywords:** SARS-CoV-2, cancer immunotherapy, MIS-C, MIS-A, COVID-19

## Abstract

Pediatric, adolescent and young adult (AYA) patients receiving novel cancer immunotherapies may develop associated toxicities with overlapping signs and symptoms that are not always easily distinguished from severe acute respiratory syndrome coronavirus 2 (SARS-CoV-2) infection/clinical sequelae. We describe 2 diagnostically challenging cases of SARS-CoV-2 and Multi-Inflammatory Syndrome-Adult (MIS-A), in patients with a history of acute lymphoblastic leukemia following cellular therapy administration and review evolving characterization of both the natural course of SARS-CoV-2 infection and toxicities experienced in younger cancer immunotherapy patients. Vigilant monitoring for unique presentations and epidemiologic surveillance to promptly detect changes in incidence of either condition may be warranted.

## Introduction

Advancements in cancer immunotherapy have led to recent approvals by the Food and Drug Administration (FDA) for chimeric antigen receptor T cell (CAR-T) and immune-checkpoint inhibitor (ICI) therapies in pediatric, adolescent, and young adult (AYA) patients with cancer (1–3). These may be associated with unique and yet poorly characterized severe immune-related adverse events. Severe acute respiratory syndrome coronavirus 2 (SARS-CoV-2) continues to pose a global health crisis, and its clinical characterization is yet to be defined. The presentation in children has been generally mild and/or atypical (4). According to the *COVID-19 in Pediatric Cancer Global Registry Data*, almost half of the 924 SARS-CoV-2 positive cases in 41 countries had a cancer diagnosis of acute lymphoblastic leukemia (ALL) and 5.7% were stem cell transplant (SCT) recipients (5). Overall, 42.3% of reported patients were asymptomatic at diagnosis and approximately 5% experienced critical disease, defined as the presence of organ dysfunction or death due to COVID-19 (5).

By May 2020, multisystem inflammatory syndrome in children (MIS-C) was characterized by shock, cardiac dysfunction, abdominal pain, markedly elevated inflammatory markers and positive SARS-CoV-2 serology or laboratory or epidemiologic evidence of infection (6, 7). In September 2020, favorable outcomes were described among SCT and CAR recipients who experienced SARS-CoV-2 infection (8). In October 2020, an illness referred to as multisystem inflammatory syndrome in adults (MIS-A) was described (9).

Because young cancer immunotherapy patients may have a mild/asymptomatic initial presentation with SARS-CoV-2, associated clinical sequelae may escape detection. The emerging and yet unknown characterization of the toxicities associated with cancer immunotherapies as well as SARS-CoV-2 illness, with potentially overlapping signs/symptoms, poses a diagnostic challenge.

## Materials and Methods

We describe (with patients’ consents) SARS-CoV-2 and MIS-A, in two patients with a history of ALL following cancer immunotherapies. SARS-CoV-2 qualitative reverse-transcriptase polymerase chain reaction (RT-PCR) developed for the Roche COBAS 6800 system was used for nasal swabs (10). Nasopharyngeal (NP) respiratory PCR utilized a FDA approved real time PCR methodology that detects the pathogens’ nucleic acid.The SARS-CoV-2 test which utilizes target enriched multiplex PCR technology to detect SARS-Cov-2 virus RNA in bronchoalveolar lavage (BAL), was used (11). The SARS-CoV-2 IgG assay test developed by Abbott Laboratories, Inc. was also utilized (12).

## Results

### Case 1

An adolescent 16-year-old male with a history of refractory B-ALL received CD-19 CAR-T therapy (tisagenlecleucel). Pre- CAR-T therapy NP swab for SARS-CoV-2 was negative. His post-CAR-T course was complicated by grade 3 cytokine release syndrome (CRS) for which he received tocilizumab once. He was discharged from the hospital 2 weeks after CAR T infusion and achieved complete ALL remission.

On day +70 post-CAR-T, he experienced intermittent headaches for two days that resolved with acetaminophen at home. One week later, he reported nasal congestion, persistent intermittent headaches and generalized fatigue for few days. He denied exposure to SARS-CoV-2 or fever. Physical examination was at baseline and laboratory results were significant for transaminitis. One week later (day +84) he developed mild cough with no associated fever or dyspnea. Respiratory PCR and NP swab for SARS-CoV-2 were negative, and his chest x-ray showed sub segmental atelectasis. Few days later, he reported tactile fever and malaise. He was afebrile in clinic, well-appearing and showed improvement of transaminitis. On day +95, he was hospitalized due to a productive cough now accompanied by dyspnea, headache, dizziness and fever. Repeat respiratory PCR and NP testing for SARS-CoV-2 were negative, and a computed tomography (CT) chest showed bilateral multifocal pneumonia and suggestion of a reverse halo sign. SARS-CoV-2 IgG and IgM antibodies were negative (the patient had ongoing B cell aplasia, consistent with CAR therapy response). He underwent BAL and received broad spectrum empiric antimicrobial therapy given his immune compromised status and intravenous immunoglobulin (IVIG) for hypogammaglobulinemia. A SARS-CoV-2 test result was not obtained from this BAL. He required oxygen supplementation with high flow nasal cannula and bilevel positive airway pressure for progressive dyspnea/hypoxemia. Repeat CT chest on day +117 showed significant improvement of bilateral lung opacities but now with new opacities within the left upper lobe. Lung biopsy showed interstitial pneumonitis. He clinically improved and was afebrile, with no oxygen requirement for 8 days prior to discharge.

**Figure 1:**
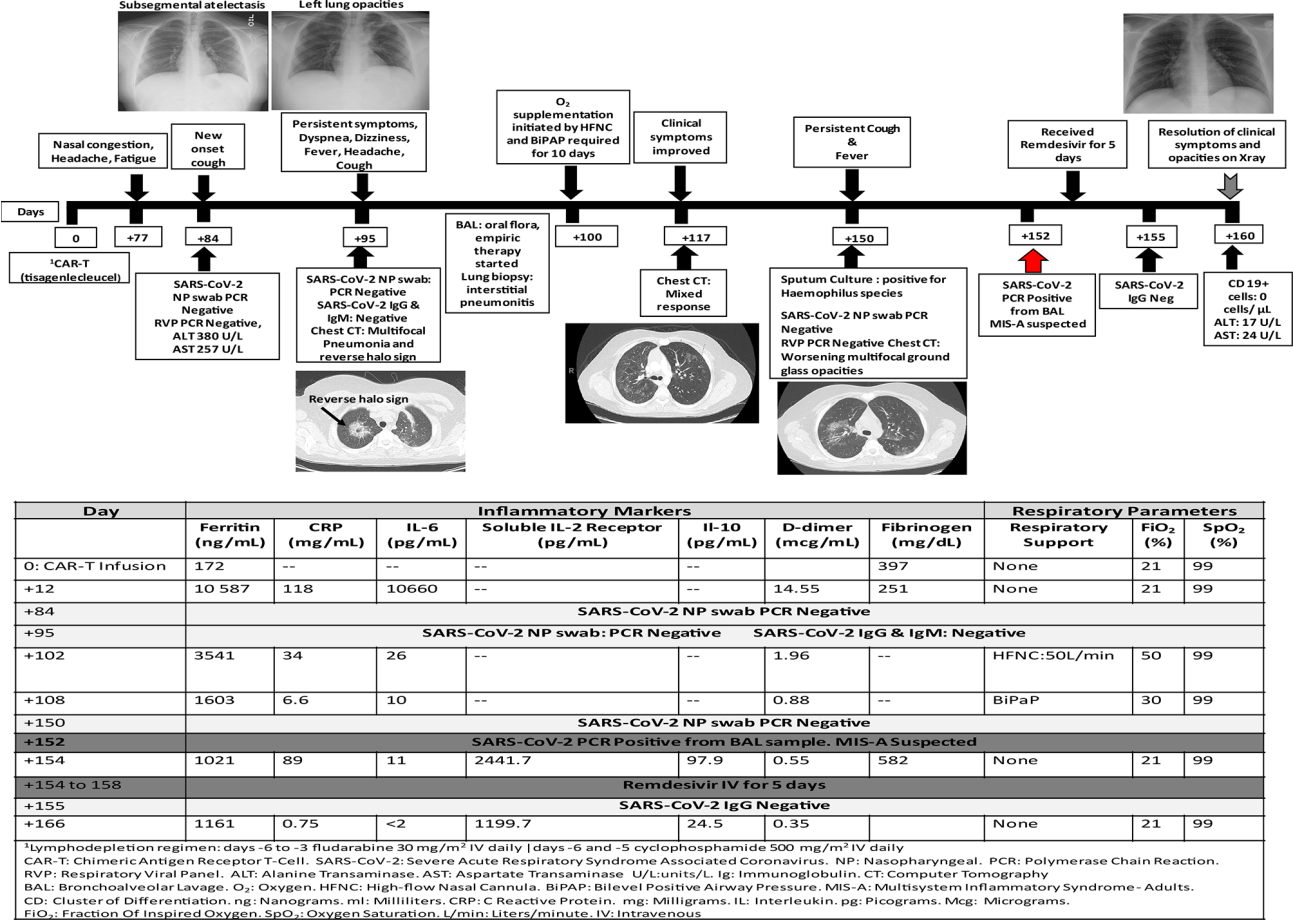
Timeline for case 1 presentation in days post-chimeric antigen receptor T-cell therapy.

On day +150 he was re-admitted for evaluation of fever and persistent cough with CT chest showing interval worsening of bilateral multifocal pneumonia. Respiratory PCR and SARS-CoV-2 NP testing were again negative. A repeat BAL was positive for SARS-CoV-2. The risks and benefits of convalescent plasma (CP) were discussed, and the patient/caregiver consented to receive this therapy on a compassionate use basis. However, during his first infusion on day +154, he developed a possible type I hypersensitivity reaction and this treatment was aborted and the family declined further treatment with CP. Given his poor humoral immunity with B-cell aplasia, he received five doses of remdesivir (200 mg IV on day 1, followed by 100 mg IV every 24 h on days 2–5) on days +154 to +158 without adverse events. He also received prophylactic dose low molecular weight heparin 0.5mg/kg on days +154 to +160. He was discharged on day +160 in good clinical condition and his most recent chest x-ray is normal. His clinical course and laboratory parameters are summarized in **Figure 1**. Unfortunately, he developed CD19+ ALL relapse on day +179.

### Case 2

A 24-year-old male with a history of relapsed B-ALL received CD19 CAR-T therapy (tisagenlecleucel), which was complicated by grade 3 CRS and grade 2 immune effector cell associated neurotoxicity syndrome (ICANS) with temporally associated atrial fibrillation. He received tocilizumab and dexamethasone and all symptoms resolved. Unfortunately, his post-CAR-T therapy disease assessment showed CD19 negative marrow disease. He achieved clinical remission with salvage therapy and subsequently underwent related haploidentical peripheral blood SCT (139 days post-CAR-T therapy). Pre-SCT NP swab for SARS-CoV-2 PCR was negative. His transplant course was complicated by very severe hepatic sinusoidal obstructive syndrome (SOS) treated with defibrotide with multi-organ dysfunction. He was discharged on day +39 in stable condition.

**Figure 2:**
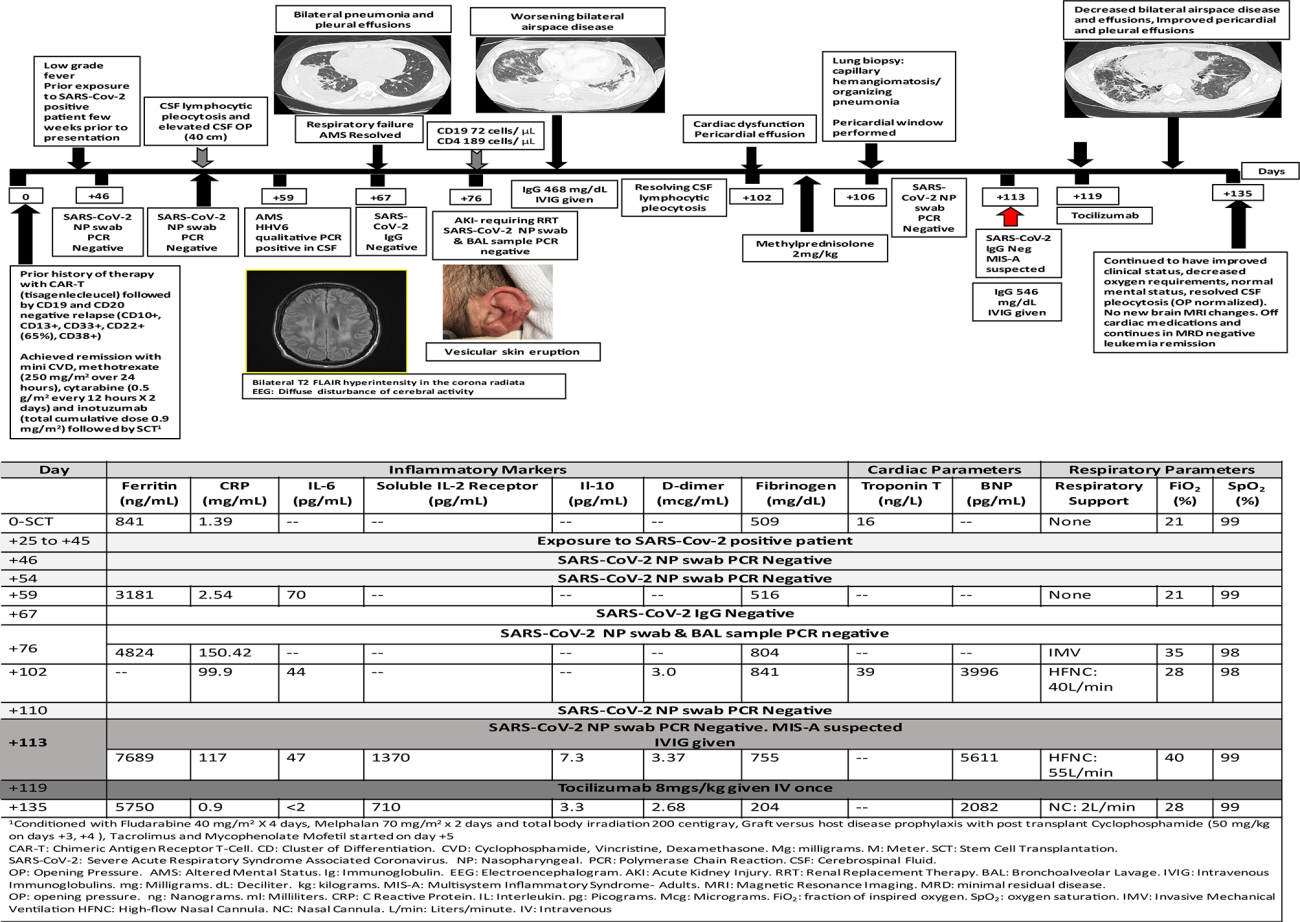
Timeline for case 2 presentation in days post-stem cell transplantation.

On day +43 post-SCT, he presented to clinic with a papular pruritic rash over the scalp, ears, neck and upper extremities that progressed to vesicular eruption (**Figure 2**). A few days later, he developed low-grade fever and received empiric anti-microbials. As part of his leukemia evaluation, cerebrospinal fluid (CSF) analysis was performed and showed elevated white blood cells (74 cells/microliter) with polymorphous lymphocytosis (no leukemia). Infectious studies were negative. On day +59 he developed confusion and delirium and was transferred to the ICU. Repeat CSF analysis showed polymorphous lymphocytosis (111 cells/microliter) and human herpes virus-6 (HHV6) was detected by qualitative but not quantitative PCR; Brain MRI showed bilateral T2 FLAIR hyperintensity in the corona radiata suspicious for leukoencephalopathy. He received empiric therapy with foscarnet for HHV6. His mental status progressively improved and returned to baseline by day +66.

On day +67, he developed acute respiratory distress with bilateral pneumonia and pleural effusions. He subsequently required mechanical ventilation on day +71. BAL samples were negative for infection. He was extubated on day +81 to high flow nasal cannula. He developed non-oliguric acute kidney injury and fluid overload that required renal replacement therapy (days +76 to +84).

Lung biopsy on day +106 showed capillary hemangiomatosis/organizing pneumonia. He developed cardiac diastolic dysfunction with dilated left ventricle, moderate pericardial effusion (required pericardial window on day +106) and elevated serum troponin-T and elevated galectin-3 (37.4 ng/ml). He required milrinone from days +116 to +125, with an ejection fraction of 55–60% and elevated BNP-max level 9165 pg/ml.

Nasal swab and bronchoscopy evaluations were repeatedly negative including SARS-Cov-2 PCR and antibody testing, and the patient did not initially endorse known exposures. Around week 9 of his illness, the patient acknowledged a household exposure approximately 2 weeks prior to the initial onset of symptoms. Review of his clinical history showed multi-system inflammation affecting his brain, kidney, heart, and lungs with increased inflammatory markers and some response to administration of methylprednisolone. With a presumed diagnosis of MIS-A he received (IVIG 0.5g/kg on day +113) and anti-cytokine therapy (tocilizumab 8mg/kg once on day +119) with gradual improvement of his multi-organ dysfunction. He also received prophylactic dose low molecular weight heparin 0.5mg/kg on days +117 to +135. He is currently weaned to nasal cannula with no cardiac and renal support and improved inflammatory markers. His clinical course and laboratory parameters are summarized in **Figure 2**.

## Discussion

**Figure 3:**
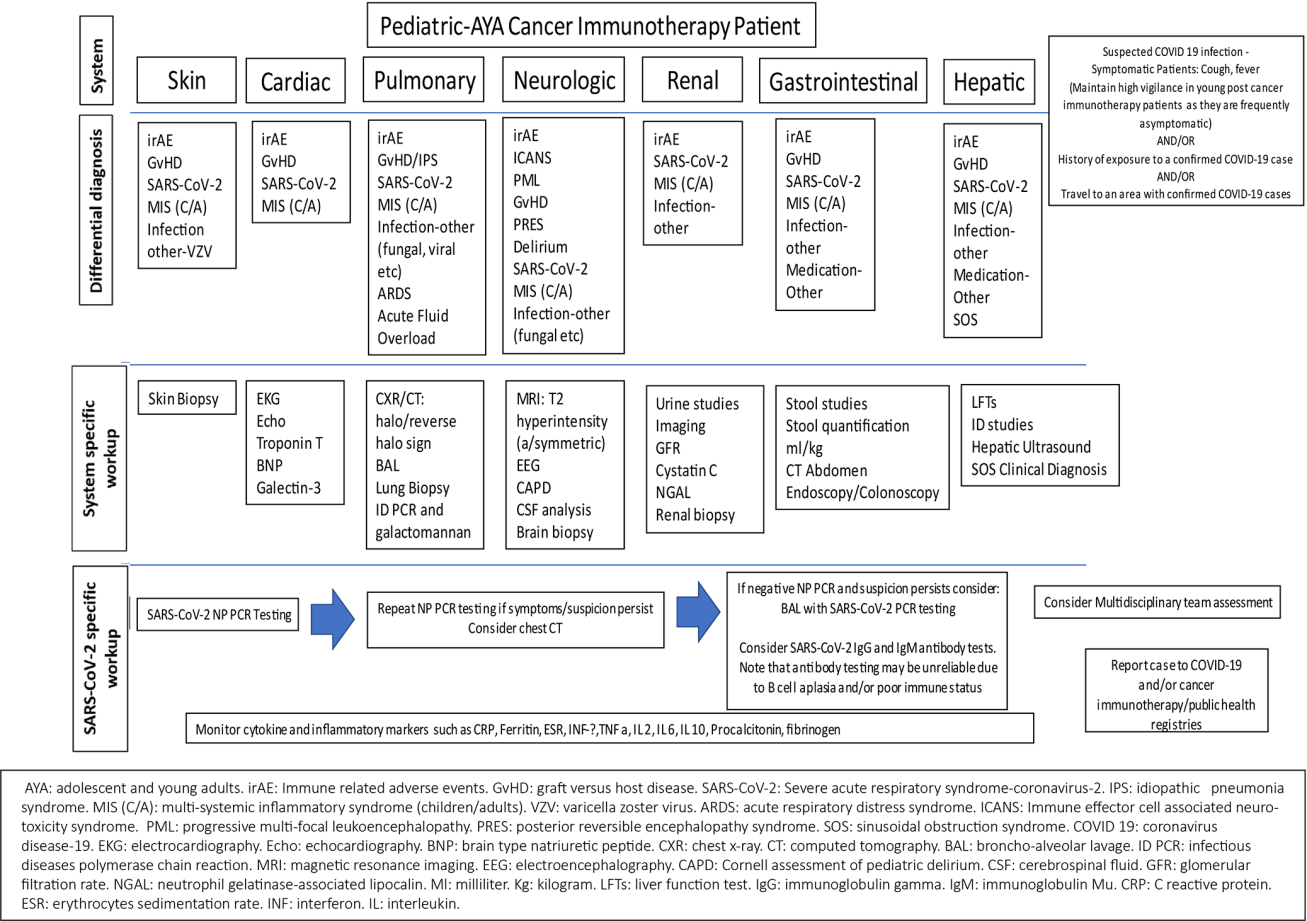
Considerations for differentiation of toxicities associated with cancer immunotherapies, SARS-CoV-2 infection, and multisystemic inflammatory syndrome in children and adults (MIS-C/A) secondary to SARS-CoV-2 infection.

The natural history and characterization of clinical sequelae of the SARS-CoV-2 in specific sub-populations, such as children and those receiving cancer immunotherapies, continue to be described. As depicted in **Figure 3**, differentiation of immune-related adverse events, such as CRS, ICANS, and COVID-19 infection *versus* associated multi-inflammatory syndrome remain a challenge. Multi-disciplinary assessments may facilitate optimized treatment decisions in challenging cases.

In a meta-analysis of 11 studies that included 3,442 respiratory samples, the reported SARS-CoV-2 detection rate was 54% (95% CI: 41–67%) for NP swabs, 43% (95% CI: 34–52%) for oropharyngeal swabs and 71% (95% CI: 61–80%) for sputum. The rate of detection was highest when performed between 0 and 7 days from the onset of symptoms and declined with time. For NP sampling, the estimated percentage positive was 80% (95% CI: 66–91%), 59% (95% CI: 53–64%) and 36% (95% CI: 18–57%) at 0–7 days, 8–14 days, and >14 days after symptom onset, respectively (13, 14). Therefore, children and cancer immunotherapy patients with atypical presentations or mild initial presentations may escape SARS-CoV-2 detection based on delayed presentation for testing. Higher rates of SARS-CoV-2 detection and viral loads have been observed in lower respiratory samples, such as BAL fluid and endotracheal aspirates, possibly due to the higher density of the SARS-CoV-2 viral receptor, the human angiotensin-converting enzyme 2 (ACE-2) receptor (15–17). For patients with a high clinical suspicion for COVID-19 who test negative for SARS-CoV-2 using upper respiratory tract specimens, the World Health Organization recommends retesting using lower respiratory samples (18). Patient 1 was tested 14 days after initial presentation of mild, atypical symptoms and repeated negative NP testing along with his initial clinical improvement could have been falsely reassuring. It is possible that this patient’s B-cell aplasia and therefore insufficient production of protective antibodies may have contributed to the delayed clearance of this virus or reinfection (19). The benefit of convalescent plasma in this scenario remains to be determined.

Immune related adverse events (irAEs) following cancer immunotherapy and MIS (C/A) may have overlapping clinical symptoms with increased inflammatory biomarkers that may play an important role in morbidity and mortality and needs further controlled studies. Common irAEs include rash, fatigue, nausea, diarrhea, thyroid abnormalities and pneumonitis. Multi-organ dysfunction may also occur post-SCT among patients with SOS, endotheliopathies, and infection. While pulmonary findings are thought to be less common in MIS (C/A), it is important to note that CT findings of cryptogenic organizing pneumonia, non-specific interstitial pneumonia, hypersensitivity pneumonitis, bronchiolitis, pleural effusions, and reverse halo sign, may also be seen with immune-related pulmonary toxicity. The reverse halo sign appears as a central ground-glass opacity surrounded by a denser region of consolidation and is distinct from the halo sign associated with pulmonary hemorrhage typically seen in angioinvasive aspergillosis in immunocompromised patients (20). Abnormal T2 hyperintensities and mental status changes may be seen in ICANS, posterior reversible encephalopathy syndrome, progressive multifocal leukoencephalopathy, and perhaps, in MIS. Increased cardiac enzymes, myositis and arrhythmias, renal dysfunction, encephalitis, hypophysitis, and colitis similarly may be observed in both conditions.

A known history of SARS-CoV-2 infection or exposure despite negative NP/antibody testing may help differentiate between conditions. MIS (C/A) may result from immune mediated injury and hyper inflammation triggered by SARS-CoV-2. Aberrant immune responses after cancer immunotherapy or SARS-CoV-2 infection (and possibly after vaccination in the future) may be observed. Vigilant monitoring for novel cancer immunotherapy toxicity and SARS-CoV-2 clinical presentations, with reporting to health department and registries is important.

Currently there are no universally accepted guidelines on the management of SARS-CoV-2 infection or MIS (C/A) with over 2,000 interventional studies for the treatment of COVID 19 registered on clinicaltrials.gov. Common recommendations for both COVID 19 and MIS (C/A) include a multidisciplinary team approach that includes the involvement of critical care, infectious disease, hematology, cardiology, rheumatology as well as immunology teams. Other recommendations include vigilant supportive care including fluid resuscitation with intravenous fluids and vasopressors if hypotension is present, cardiac monitoring, and anti-coagulation. Treatment guidelines also include the use of antivirals such as remdesivir for patients who are in the acute phase of illness where the virus is actively replicating, corticosteroids and other immunomodulatory agents such as tocilizumab.

Corticosteroids have been recommended in both SARS-CoV infection and subsequent MIS (C/A). In the UK RECOVERY trial—a randomized controlled trial of over 6,000 patients hospitalized with COVID-19, 2104 patients received dexamethasone in addition to standard of care (SOC) and 4,321 received SOC only. The use of dexamethasone resulted in a lower 28 day mortality among those requiring invasive mechanical ventilation (29.3 *vs*. 41.4%; rate ratio, 0.64; 95% CI, 0.51 to 0.81) and among those receiving oxygen without invasive mechanical ventilation (23.3 *vs*. 26.2%; rate ratio, 0.82; 95% CI, 0.72 to 0.94) but not among those who did not require respiratory support (21). While other studies have also shown a benefit of corticosteroids on the short term mortality from patients hospitalized with COVID-19, the optimal initiation, dose, duration, and indication for corticosteroids require further studies (21–24). For MIS (C), IVIG (2g/kg) and glucocorticoids (1–2mg/kg/day) have been recommended as the first line therapy in children and this treatment has also been used in adults (9, 25).

Tocilizumab (TCZ) an IL-6 receptor antagonist has also been used as a potential treatment in patients with COVID-19 due to its ability to block the IL-6 mediated inflammatory response. In a meta-analysis of 16 studies and 3,641 patients, there was a statistically significant reduction in the mortality of patients with severe COVID19 when treated with TCZ, but this difference was not significant if patients received corticosteroids. Given the heterogeneity across studies however, it is difficult to draw any firm conclusions regarding the use of TCZ from this meta-analysis (26). Given its ability to decrease the cytokine storm TCZ has also been included for consideration for the treatment of refractory or severe MIS (C/A) (27). Similarly anakinra an IL-1 receptor blocker may also be considered in severe or refractory cases of MIS (C/A) (27).

In both COVID 19 infection and MIS (C/A) there have been reports of hypercoagulability and increased risk of thrombosis leading to the recommendation of prophylactic dosed anticoagulation-low molecular weight heparin by the National Institute of Health, European Group for Blood and Marrow Transplantation (EBMT) and American College of Rheumatology (AACR) (13, 25, 28). Given its similarity to Kawasaki disease, antiplatelet agents such as aspirin have also been recommended in patients with MIS (C/A) (25).

Of note, the effectiveness and safety of the above mentioned treatments still remain to be determined by further studies and large scale randomized controlled trials. Furthermore the efficacy of the much anticipated COVID-19 vaccination remains to be determined in patients who are post-hematopoietic cellular therapy (HCT). In the interim, multiple other promising therapies continue to be evaluated including the use of favipravir (antiviral), infliximab (TNF α monoclonal antibody), convalescent plasma and baricitinib (janus kinase inhibitor) and anti-SARS-CoV-2 monoclonal antibodies bamlanivimab and casirivimab plus imdevimab (28–31). As the data surrounding COVID19 infection and MIS (C/A) continue to rapidly evolve the above mentioned treatment and management strategies are subject to change as data emerges. A broad overview of available treatments is summarized in **Table 1** (25, 28, 32–34).

**Table 1:** Brief overview of current treatment options available for COVID 19 and multisystem inflammatory syndrome in children and adults (14, 25, 28, 32, 33).

Drug	Indication	Notes
Remdesivir (antiviral)	Hospitalized patients with COVID19 who require supplemental oxygen	Remdesivir is the only drug FDA approved for the treatment of COVID 19. It is approved for patients ≥12 years and weigh ≥40 kg but is also available for younger children (and those weighing <40 and >3.5 kg) through an FDA EUA
Glucocorticoids (immunomodulator)	Hospitalized patients COVID19 who require supplemental oxygen First line agent for MIS (C/A)	Not specifically FDA approved for COVID 19 infection or MIS (C/A).
IVIG (immunomodulator)	First line agent for MIS (C/A)	Not specifically FDA approved for COVID 19 infection or MIS (C/A).
Tocilizumab (IL-6 receptor antagonist)	May be considered for MIS refractory to IVIG and/or glucocorticoids	Not specifically FDA approved for COVID 19 infection or MIS (C/A).
Anakinra (IL-1 receptor antagonist)		Not specifically FDA approved for COVID 19 infection or MIS (C/A).
Baricitinib (JAK inhibitor)	Available under EUA for the treatment of suspected or laboratory confirmed COVID-19 in hospitalized patients ≥ 2 years of age requiring supplemental oxygen, IMV, or ECMO, in combination with remdesivir	
Casirivimab plus imdevimab (SARS-CoV-2 neutralizing antibody)	Available under EUA for non-hospitalized patients ≥12 years of age weighing ≥40 kg with mild to moderate COVID-19 who are at high risk for progressing to severe disease and/or hospitalization	
Bamlanivimab (SARS-CoV-2 neutralizing antibody)		
**Investigational drugs/treatment**		
Anti-SARS Cov-2 T cell infusions for COVID 19 (BATIT) (SARS-CoV2-specific T cells) NCT04401410	Hospitalized COVID19 patients with high risk of progression to mechanical ventilation.	
Convalescent plasma (passive immune therapy)	Hospitalized patients with COVID19 infection, indications vary per trial	

COVID 19, coronavirus disease 2019; FDA, Food and Drug Administration; EUA, Emergency Use Authorization; Kg, Kilogram; MIS (C/A), multisystem inflammatory syndrome (children/adults); IVIG, intravenous immune globulin; IL, interleukin; JAK, Janus kinase; IMV, invasive mechanical ventilation; ECMO, extracorporeal membrane oxygenation; SARS-CoV-2, severe acute respiratory syndrome associated coronavirus.

While optimal treatment algorithms of both SARS-CoV-2 related syndromes and cancer immunotherapy adverse events continue to evolve, prompt differentiation of both should lead to initiation of definitive treatments as indicated and available. Close epidemiologic surveillance for an increase in adverse events (including late events) among cancer immunotherapy patients above historical rates during the pandemic may be warranted.

## Author Contributions

DR and SK completed the data collection and wrote the manuscript. RM, LE, J-BD, LB, JT, NDG, DP, PT, AHA, JC, SR, KM, RS, WW, LC, JG, WZ, and ND treated the patient and assisted in editing the manuscript. MB, BS, CG, WT, PK, KR, ES, and RC assisted in editing the manuscript; KM treated the patient, assisted in analyzing the data, and wrote the manuscript. All authors contributed to the article and approved the submitted version.

## Conflict of Interest

The authors declare that the research was conducted in the absence of any commercial or financial relationships that could be construed as a potential conflict of interest.
